# Dimensions of apathy in Parkinson's disease

**DOI:** 10.1002/brb3.2862

**Published:** 2023-05-18

**Authors:** Nasya Thompson, Michael MacAskill, Maddie Pascoe, Tim Anderson, Campbell Le Heron

**Affiliations:** ^1^ New Zealand Brain Research Institute Christchurch New Zealand; ^2^ Department of Medicine University of Otago Christchurch New Zealand; ^3^ Department of Neurology Christchurch Hospital, Te Whatu Ora ‐ Health New Zealand Waitaha Canterbury New Zealand

**Keywords:** apathy, dimensional apathy, non‐motor symptoms, Parkinson's disease

## Abstract

**Introduction:**

Apathy is one of the most common neuropsychiatric manifestations in Parkinson's disease (PD). Recent proposals consider apathy as a multidimensional construct, which can manifest in behavioral, cognitive, emotional, and/or social dimensions. Apathy also overlaps conceptually and clinically with other non‐motor comorbidities, particularly depression. Whether all of these dimensions are applicable to the apathetic syndrome experienced by people with PD is unclear. In the present study, we investigated the multidimensional pattern of apathy associated with PD, using the recently developed Apathy Motivation Index (AMI) which probes behavioral, emotional, and social apathy dimensions. We then examined the relationship between these dimensions and other features of PD commonly associated with apathy, including depression, anxiety, cognition, and motor state.

**Methods:**

A total of 211 participants were identified from the New Zealand Brain Research Institute (NZBRI) longitudinal PD cohort. One hundred eight patients and 45 controls completed the AMI, administered as an online questionnaire, and additional assessments including neuropsychiatric, neuropsychological, and motor scores. The pattern of dimensional apathy in PD was assessed using a repeated‐measured analysis of variance, while simple linear regressions were performed to evaluate relationships between these dimensions and other variables.

**Results:**

We found a significant interaction between group (PD versus control) and apathy subscale, driven mainly by higher levels of social and behavioral—but not emotional—apathy in those with PD. This result was strikingly similar to a previous study investigating social apathy in PD. Distinct patterns of dimensional apathy were associated with depression and anxiety, with social and behavioral apathy positively associated with depression, and emotional apathy negatively associated with anxiety.

**Conclusion:**

This work provides further evidence for a distinct pattern of apathy in people with PD in which deficits manifest in some—but not all—dimensions of motivated behavior. It emphasizes the importance of considering apathy as a multidimensional construct in clinical and research settings.

## Graphical abstract

Apathy in Parkinson's disease manifests mainly along social and behavioural, rather than emotional dimensions. Increasing social and behavioural apathy is associated with more depressive symptomatology in this group. In contrast, the presence of emotional apathy is negatively associated with anxiety, demonstrating dissociable relationships between dimensions of apathy and other common non‐motor complications of PD.

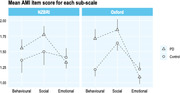

## 1 INTRODUCTION

Parkinson's disease (PD) is a common neurodegenerative disorder that causes widespread physiological and anatomical disturbances in which dopaminergic deafferentation of the basal ganglia is a primary feature (Alexander, 2004; Galvan & Wichmann, 2008). While PD was traditionally perceived as a paradigmatic movement disorder, with characteristic motor symptoms of tremor, rigidity, and bradykinesia, neuropsychiatric symptoms are increasingly recognized as some of the most debilitating and complex aspects of the disease (Chaudhuri et al., 2015; Chaudhuri, Healy, & Schapira, 2006; Prakash, Nadkarni, Lye, Yong, & Tan, 2016). Apathy is one of the most common neuropsychiatric manifestations in PD, estimated to be present in 30%–40% of patients (Chase, 2011); however, it is often overshadowed in clinical assessments by the more overt motor symptoms, leading to under‐recognition and undertreatment (Shulman, Taback, Bean, & Weiner, 2001; Sullivan, Ward, Hauser, & Zesiewicz, 2007).

Apathy has been conceptualized as a diminution of motivation and interest that manifests as a deficit in goal‐directed behavior (Marin, 1991; Starkstein, Brockman, & Hayhow, 2012). Its presence is associated with significantly diminished quality of life, increased levels of disability, and increased carer burden (Benito‐León, Cubo, Coronell, & Group, 2012; den Brok et al., 2015; Leroi et al., 2012). Apathy can occur in many brain disorders (Le Heron, Holroyd, Salamone, & Husain, 2019). Recent proposals consider apathy as a multidimensional construct, which can manifest in behavioral, cognitive, emotional, and/or social dimensions (Le Heron et al., 2019; Radakovic, Davenport, Starr, & Abrahams, 2018). However, it is still unclear whether all of these dimensions are applicable to the apathetic syndrome experienced by people with PD. Furthermore, although various self‐report and clinician‐administered questionnaires have been developed to assess these purported dimensions of apathy, including the Lille Apathy Rating Scale and the Dimensional Apathy Scale, these measures do not explore how amotivation may manifest within the social domain—an increasingly recognized and dissociable apathy component (Ang, Lockwood, Apps, Muhammed, & Husain, 2017; Ang et al., 2018). To address this problem, the Apathy Motivation Index (AMI) was developed recently. The AMI dissects apathy into social (the level of engagement in social interactions), behavioral (the tendency to self‐initiate goal‐directed behavior), and emotional (feelings and/or responses of affection) domains and has been rigorously validated in a large sample of healthy individuals (Ang et al., 2018).

Recently, a single center study in which people with PD completed the AMI revealed a pattern of apathy characterized by impaired behavioral and social—but not emotional—motivation compared to healthy controls (Ang et al., 2018), but these results have not yet been replicated. This is important given that this pattern of changes differed from previous work using alternative questionnaires. Using the Dimensional Apathy Scale, Santangelo et al. found that behavioral initiation and emotional—but not executive—subscales of apathy were altered in PD compared to controls. Application of the Lille Apathy Rating Scale to people with PD found the most marked differences from healthy controls on action initiation and intellectual curiosity subscales, with additional differences on emotional and self‐awareness subscales in those with PD dementia (Dujardin et al., 2007; Santangelo et al., 2017). Thus, there remains some discrepancy—complicated by the use of different questionnaires whose named subscales may in fact not be directly comparable—about the exact patterns of apathy associated with PD.

While apathy is a discrete entity in PD, it also overlaps conceptually and clinically with other non‐motor comorbidities, particularly depression. These overlaps may be best understood within a broader neurocognitive framework for apathy, whereby specific disruptions of cognitive processes—such as aspects of reward processing—could lead to both loss of motivation and depressive symptomatology such as anhedonia (Costello et al., 2022). However, questionnaire subscales also offer a more nuanced understanding of the relationship between aspects of apathy and depression (Ineichen & Baumann‐Vogel, 2021), and also the lesser investigated association between apathy and anxiety (Ineichen & Baumann‐Vogel, 2021; Maillet et al., 2016). Additionally, although motor severity and cognitive dysfunction are often associated with apathy in PD (Aarsland et al., 2009), less is known about how these associations may vary between dimensions of apathy. Overall, delineating the relationship between the distinct profiles of apathy and other neuropsychiatric and cognitive features in PD is critical for developing a richer understanding of these important non‐motor features of PD, linking the different clinical phenotypes of amotivation to underlying systems disruptions, and ultimately targeting interventions.

In the present study, we investigated the multidimensional pattern of apathy associated with PD, using the recently developed AMI. Based on previous work, we hypothesized that people with PD would have higher levels of behavioral and social—but not emotional—apathy than controls. We then examined the relationship between these apathy dimensions and other features of PD commonly associated with apathy, including depression, anxiety, cognition, and motor state. We hypothesized that the pattern of dimensional apathy would vary between these distinct features of PD.

## 2 MATERIALS AND METHODS

### 2.1 Participants

A total of 211 participants were identified from the New Zealand Brain Research Institute (NZBRI) longitudinal PD cohort. Full details of this cohort are published elsewhere (MacAskill et al., 2022; Wood et al., 2016), but all included patients had been diagnosed with PD by a movement disorder trained neurologist, in accordance with Movement Disorders Society criteria. All participants provided written informed consent, and the longitudinal study is approved by the Health and Disability Ethics Committees of the Ministry of Health.

### 2.2 Assessments

Participants were invited via email to complete the AMI, administered as an online questionnaire using REDCap, a secure web application. To maximize the response rate, a further reminder email was sent out 1 week after the initial request, and we then followed up with phones calls 2–3 weeks later for those who had not yet responded. The AMI is a multidimensional scale composed of 18 items constituting three subscales assessing behavioral, social, and emotional apathy. Each item was scored from 0 to 4, with a higher score indicating increased severity of apathy. Cut‐offs for clinically significant apathy were based on previous publications of this scale, and set as > 1 standard deviation above the mean (17). Full AMI is available in Supporting Information. Data collection occurred a few months following a period of nationwide lockdown during the COVID‐19 pandemic.

Additional assessments were available from the ongoing PD longitudinal study including neuropsychiatric, neuropsychological and motor scores (30). In particular, these included the following: (i) Neuropsychiatric Inventory (NPI) (31), (ii) Hospital Anxiety and Depression Scale (HADS) (32), (iii) Montreal Cognitive Assessment (MoCA) (Nasreddine et al., 2005), and (iv) MDS‐UPDRS part III.

### 2.3 Statistical analysis

Data were analyzed using R version 4.2.1 (33). Dimensions of apathy were assessed using a repeated measures analysis of variance with participant group (patient or control) as the between‐subject factor and AMI subscales (behavioral, social, emotional) as within subject factors, followed by pairwise comparisons for each subscale. The association between AMI score and the presence of apathy defined by NPI apathy subscale score (greater than or equal to 2) was assessed using an unpaired Welch's *t*‐test.

To analyze the relationship between apathy and each of depression, anxiety, cognition, and motor score, we performed independent simple linear regressions with AMI subscore as the dependent variable. Between‐group comparison of demographic and clinical data was performed by parametric test statistic (Welch's *t*‐test) and chi‐square test as appropriate.

**FIGURE 1:**
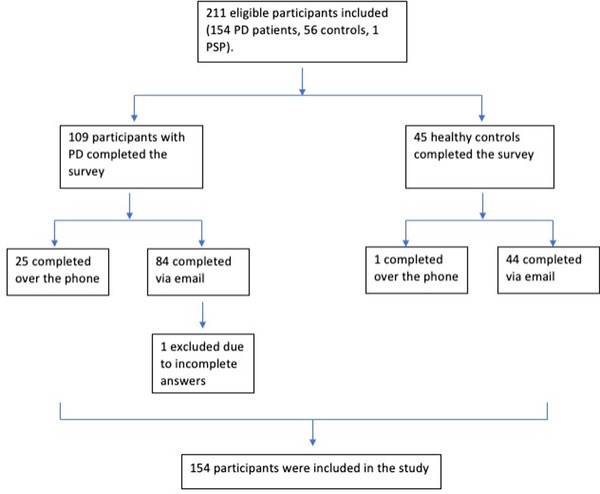
CONSORT trial flow diagram

Of the 211 participants invited to complete the AMI questionnaire, 129 responded via email and 25 completed the survey over the phone. One respondent was excluded due to incomplete answers (Figure 1). Thus, 153 participants were included: 45 healthy controls and 108 patients with PD. Table 1 summarizes the baseline descriptive demographic profiles, including age, sex, education, cognitive status, and UPDRS motor scores of the participants in these two groups. The control participants were significantly older than those in the PD group, while other demographic variables were broadly similar across both groups, although the PD group had a significantly lower average MoCA score than controls. Of the participants with PD, 102 (94%) were taking anti‐parkinsonian medication. Of those, the mean levodopa‐equivalent daily dose was 979 mg (SD 501, range 50–2525). The number and proportion of those taking specific medications or classes of medications can be seen in Table 2. One patient had undergone subthalamic nucleus deep brain stimulation, and this was in the ‘‘ON’’ phase during assessments.

**TABLE 1:** Demographics of study sample

Demographics	Control	PD	Group difference
Number	45	108	
Age	77.5 (7.3)	72.6 (6.9)	*p* <.001
Female	44.4%	35.2%	*p* = .4
Years of education	13.9 (2.6)	13.0 (2.6)	*p* = .06
MoCA	27.0 (2.5)	25.0 (3.4)	*p* <.001
MDS‐UPDRS III	7.3 (7.9)	32.9 (13.7)	*p* <.001

*Note*: Mean and standard deviation reported where appropriate. Significance was assessed using Welch's t‐test for age, education and UPDRS Part III and Chi squared analyses for sex.

Abbreviations: MoCA, Montreal Cognitive Assessment; PD, Parkinson's disease.

**TABLE 2:** Anti‐parkinsonian medications utilized in patients with Parkinson's disease (PD). This table outlines the usage of medications across the whole sample and when breaking up in to those with normal Apathy Motivation Index (AMI) and abnormal AMI scores (i.e., moderate to severe)

Medication	All PD	AMI normal	AMI abnormal
Levodopa	99 (92%)	79 (91%)	20 (95%)
Dopamine agonists	60 (56%)	50 (57%)	10 (48%)
Amantadine	45 (42%)	33 (38%)	12 (57%)
Selegiline	25 (23%)	21 (24%)	4 (19%)
Anticholinergics	6 (6%)	5 (6%)	1 (5%)
Apomorphine	1 (1%)	0 (0%)	1 (5%)

## 3 RESULTS

### 3.1 Variation in dimensions of apathy in Parkinson's disease

**FIGURE 2:**
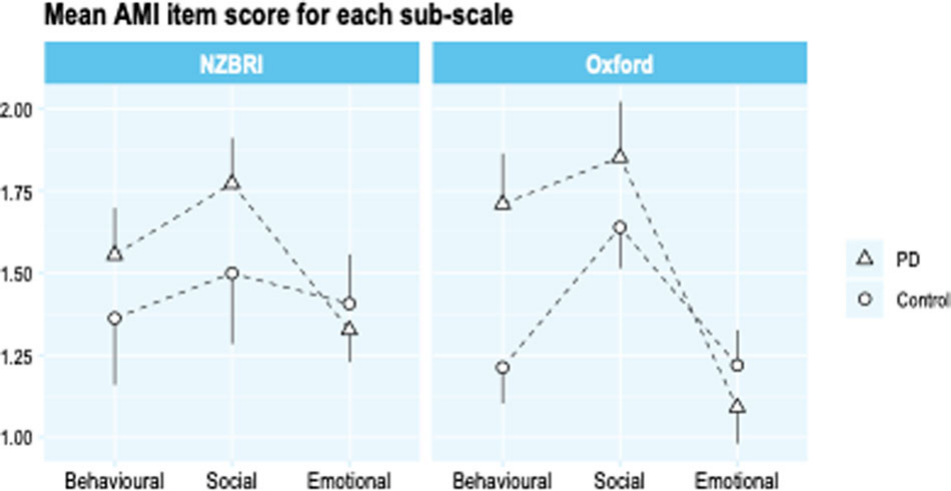
Comparison of apathy dimensions between people with Parkinson's disease (PD) and controls. The left panel shows the current cohort (NZBRI), while the right panel shows results from a previous study (Ang et al., 2018) for comparison. In the NZBRI cohort, there was a significant interaction between group and subscale, driven mainly by participants with PD having significantly greater levels of social apathy, and a trend toward greater levels of behavioral apathy. Emotional apathy levels were very similar between the two groups. The NZBRI results demonstrate a very similar pattern to those obtained in the prior Oxford study. Graph represents the mean sub score ± SE. significance was assessed using a mixed model analysis of variance. *p <.05

There was a significant interaction between group and subscale (*F*(2,302) = 3.56, *p* = .045) (Figure 2, left panel). Post hoc analysis suggested that this was driven mainly by patients with PD having significantly higher social apathy scores (mean difference (md) = 1.6, *t*(84) = 2.1, *p* = .036), as well as non‐significantly higher behavioral apathy (md = 1.2, *t*(90) = 1.6, *p* = .12). Emotional apathy scores were similar relative to controls (md = −0.5, *t*(85) = 0.9, *p* = .37). There was no significant main effect of group (healthy controls vs. patients with PD) on total AMI score (*F*(1,151) = 2.23, *p* = .14). There was a significant main effect of subscale (*F*(2,302) = 7.77, *p* = .002). Overall, the pattern of results was very similar to that observed in the previous (Oxford) study (Figure 2, right panel).

**FIGURE 3:**
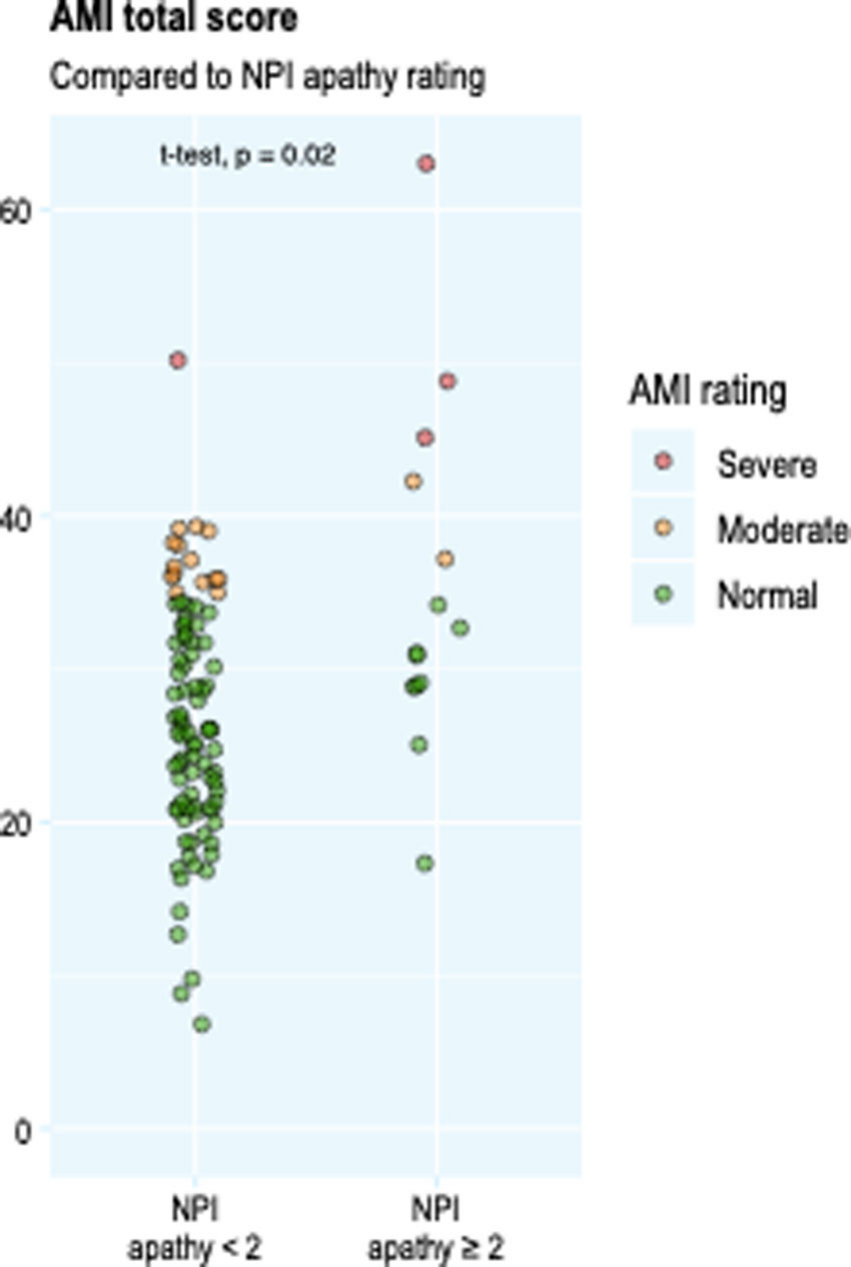
Higher Apathy Motivation Index (AMI) scores in those classified as apathetic by Neuropsychiatric Inventory

Using the cut‐off scores for moderate apathy as proposed by Ang et al. (2017), 21 of the 108 participants with PD (and five out of 45 healthy controls) were classified as apathetic based on the AMI total score. When taking into account the AMI subscales, 38 of the 108 patients (35%) and 13 out of the 45 healthy controls (30%) were apathetic on at least one dimension. The AMI total score was significantly higher in patients defined as apathetic based on their NPI‐apathy subscale score, compared to those defined as not apathetic (NPI‐apathy mean = 35.3, NPI‐no apathy mean = 26.7, *t*(14.8) = 2.71, *p* = .016) (Figure 3).

### 3.2 Subdomains of apathy are differentially associated with depression and anxiety

**FIGURE 4:**
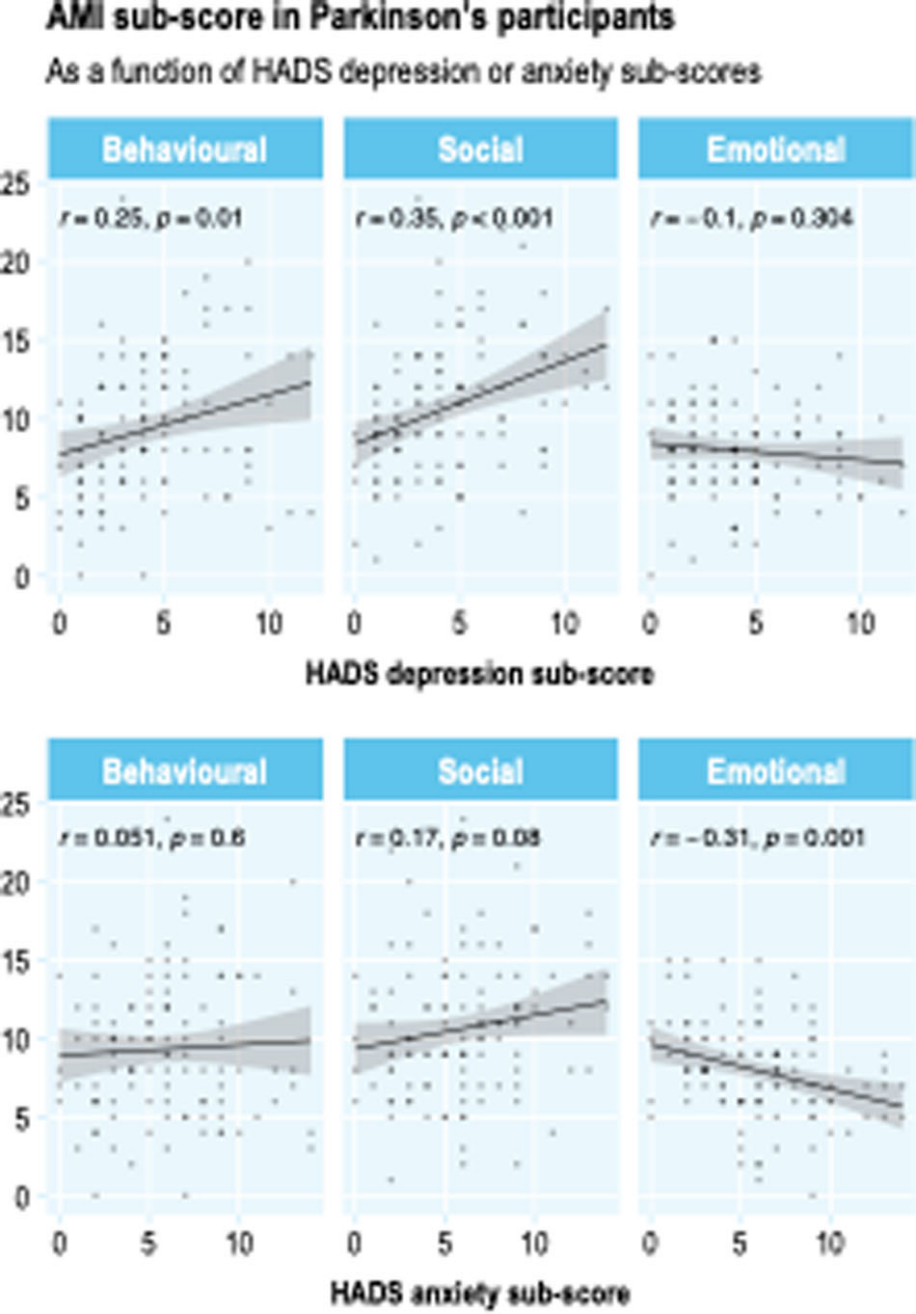
Differential association of Apathy Motivation Index (AMI) subdomains with depression and anxiety in Parkinson's disease (PD). Higher levels of behavioral and social apathy were associated with higher levels of depression, whereas higher levels of emotional apathy were associated with lower levels of anxiety.

Behavioral and social dimensions of apathy were significantly positively correlated with degree of depression (behavioral: *r* = 0.25, *p* = .01; social: *r* = 0.35, *p* <.001). There was no significant association between emotional apathy and depression score (*r* = −0.1, *p* = .30). In contrast, there was a significant relationship between emotional apathy and degree of anxiety, with higher levels of emotional apathy associated with lower levels of anxiety (*r* = −0.31, *p* = .001). Behavioral and social apathy were not significantly associated with degree of anxiety (behavioral: *r* = 0.05, *p* = .6; social: *r* = 0.17, *p* = .08, Figure 4).

### 3.3 Dimensional apathy, cognition, and motor scores

**FIGURE 5:**
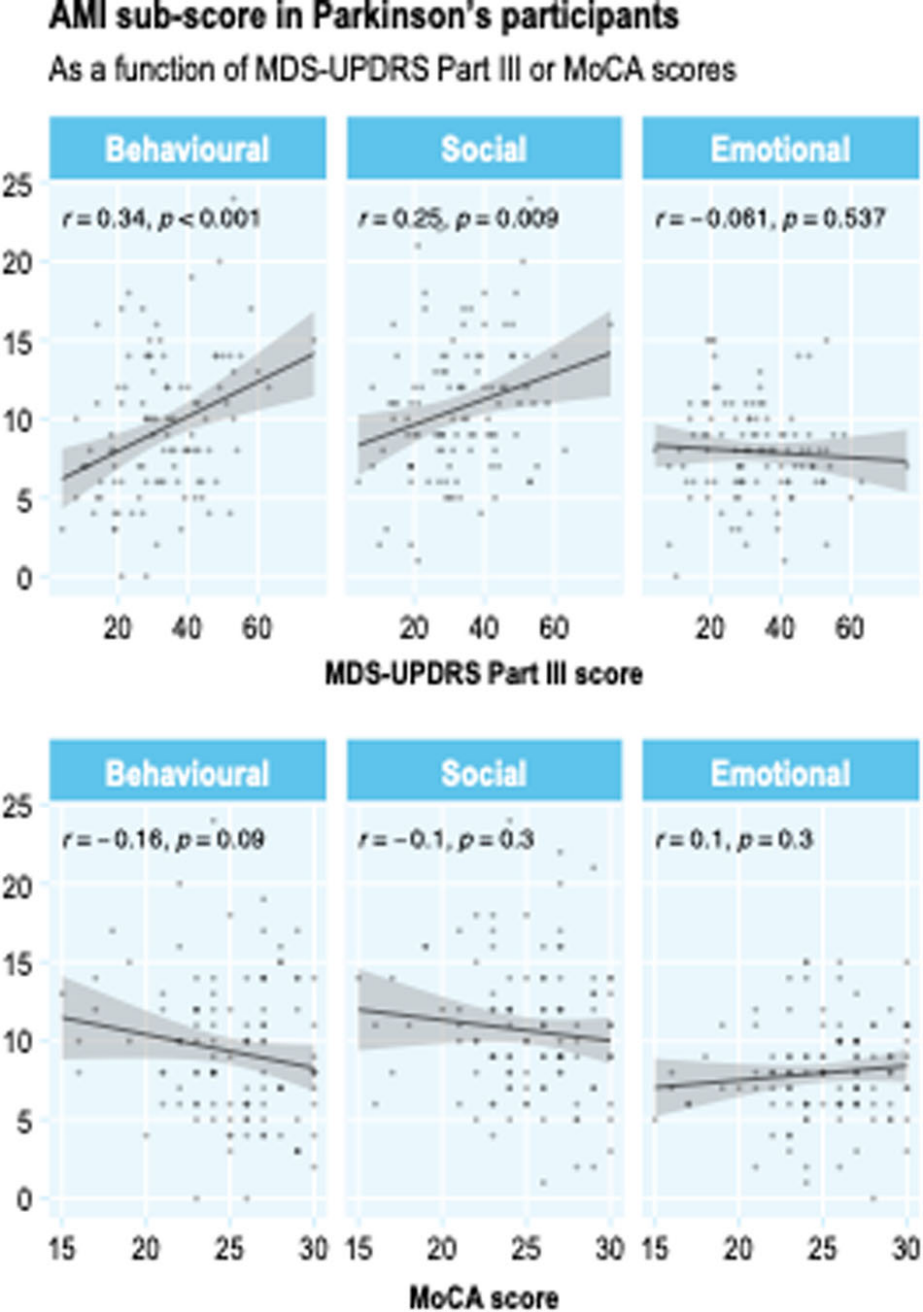
Relationship between dimensional apathy and motor severity (top panels) and cognition (bottom panels)

Overall, we did not observe a significant relationship between the dimensions of apathy and a measure of global cognition (Figure 5, bottom panels). Both behavioral apathy and social apathy—but not emotional apathy—were associated with higher scores on the UPDRS motor examination (Figure 5, top panels).

## 4 DISCUSSION

We investigated the multidimensional nature of apathy in PD, and its association with both neuropsychiatric and other features of this condition using a recently developed questionnaire (AMI) that includes assessment of the social dimension of apathy. We found that not all dimensions of motivation are impaired in PD. In particular, although levels of social and behavioral apathy tended to be higher in people with PD, levels of emotional apathy were not higher in people with PD. Furthermore, depression and anxiety were differentially associated with these different dimensions of apathy. Specifically, depression was strongly associated with both behavioral and social, but not emotional, apathy, whereas in contrast there was a strong (negative) association between anxiety and emotional apathy.

The pattern of apathy associated with PD observed in this study, characterized by greater social and behavioral, but not emotional apathy levels, was strikingly similar to what was seen in a previous study in PD to use the AMI (Y. S. Ang et al., 2018). No consensus exists as to the most appropriate method or scale to define apathy in PD (Marinus, Zhu, Marras, Aarsland, & van Hilten, 2018), but these results emphasize the importance of considering the social dimension of the apathy construct when doing so (Ang et al., 2017). Along with this earlier study, and another small study using an alternative scale (DAS) (Radakovic et al., 2018), this also provides further evidence suggesting that apathy in PD may not significantly manifest within the emotional domain. However, it should be noted that other studies have found evidence of greater emotional apathy in people with PD (Dujardin et al., 2007; Santangelo et al., 2017), and the issue remains contentious (Zigmond & Snaith, 1983). How can these discrepancies be explained? Emotion and emotional responses are a broad, multifaceted construct in themselves and have been shown to be altered in PD (Paul, Sher, Tamietto, Winkielman, & Mendl, 2020; Péron, Dondaine, Le Jeune, Grandjean, & Vérin, 2012). One possibility is that different questionnaire subscales, while labeled as probing the ‘‘emotional’’ domain, may in fact differ in what parts of the domain they are measuring—parts that may be differentially affected in PD. The emotional domain of the AMI, emphasizing emotional blunting rather than dysphoric features, probably corresponds most closely to the reward deficiency syndrome proposed by Pagonabarraga et al. (Ang et al., 2017; Pagonabarraga, Kulisevsky, Strafella, & Krack, 2015); however, further work that links direct assessments of emotional processing/responding to dimensions of apathy will be important to better understand this finding and previous findings.

Using the AMI, we found that 35% of patients with PD were clinically apathetic on at least one dimension. This finding is in line with current estimates of the prevalence of apathy in PD, which generally range from 30% to 40% depending on the assessment instrument and sample population (den Brok et al., 2015; Pagonabarraga et al., 2015). Additionally, AMI scores were significantly higher in those with apathy as assessed by the neuropsychiatric inventory, providing further evidence of convergent validity for this recently developed scale. We also observed higher than expected rates of apathy in the healthy control population (30% of controls were classified apathetic on at least one subscale). The study was undertaken 6 months after a COVID‐19‐related lockdown in New Zealand, during which time normal activities (for all people) were significantly limited. Although the most significant restrictions had been lifted at the time of questionnaire administration, it is a distinct possibility that this may have contributed to the higher than expected levels of apathy symptoms reported in our control group. In turn, this may have reduced the overall differences in apathy between patients and controls that may have otherwise been present.

Apathy is conceptualized as distinct from, but overlapping with, depression in PD (Pluck & Brown, 2002). At a mechanistic level, this overlap may correspond to impairments in aspects of reward processing and cost‐benefit decision‐making (Costello et al., 2022; Husain & Roiser, 2018). At face value, these factors seem directly related to emotional processing—which has also been conceptualized within a reward framework (Rolls, 2013). However, here, we found significant relationships between behavioral and social—but not emotional—apathy, and depression. The finding of the same pattern in previous work—both in a separate PD population and in healthy controls—suggests that this is unlikely to be a chance finding (Ang et al., 2017; Ang et al., 2018). Furthermore, previous work also related this pattern to changes in anhedonia—the component of depression thought most closely linked to apathy (Husain & Roiser, 2018). While caution must be exercised in attributing questionnaire components to labels (e.g., emotion) with strong neurobiological connotations, overall these findings provide further evidence of a more nuanced relationship between apathy and depression—a relationship that can be better understood by future work that includes dissociable dimensions of depression as well. Interestingly, emotional apathy did have a strong association in this study—but with *reduced* levels of anxiety. Apathy and anxiety are two of the most common and disabling co‐morbidities associated with PD, but the interplay between the two has not received much attention to date in the literature. Recent work has suggested that there may be a positive correlation between the occurrence of each (Ineichen & Baumann‐Vogel, 2021; Maillet et al., 2016), but here we find the opposite—those with higher levels of emotional apathy in fact were less likely to be anxious. Intuitively, this seems plausible—as emotional apathy is generally considered to involve a blunting of emotional responses to good or bad events and could be predicted to reduce levels of anxiety. However, further work is needed to disentangle the mechanisms underpinning this finding. More broadly, it suggests the emotional subscale of the AMI probes an important feature—just not one that is markedly altered as a function of either PD or associated with depression.

We did not find a significant relationship between any of the dimensions of apathy and overall cognition, assessed by the MoCA. At a group level, greater levels of cognitive impairment have been associated with apathy in PD, but it remains unclear whether this is driven by particular domains of impaired cognition (Marinus et al., 2018; Weintraub et al., 2005). Because of the relatively low level of total apathy present in the PD group in this study (∼19%), we would caution against over‐interpreting this null result, but these findings do suggest that there is no strong dimensional pattern of apathy associated with cognitive impairment in PD. In contrast, both behavioral and social apathy levels were strongly positively correlated with the degree of motor impairment present. While this could simply be a marker of disease stage, the lack of similar findings with cognition to a degree argue against this interpretation. Although the cross‐sectional nature of this study limits our ability to make causal inferences, one possible explanation for this dimensional relationship is that motor impairment impacts more significantly on behavioral and social aspects of motivated behavior simply because they are more closely related to movement. Thus, it exacerbates apathy in these dimensions relative to the emotional one. The idea of using scales that limit the influence of motor symptoms in assessing apathy has been proposed by others recently (Radakovic et al., 2018). However, even if the etiology of apathy in most people with PD relates to disrupted reward processing and decision‐making mechanisms that form the core of goal‐directed behavior (Le Heron et al., 2019), it seems plausible that greater motor disability could both increase the motor cost of such behaviors, as well as leading to more socially impoverished environments, which could in turn amplify apathetic traits.

The present study has some limitations. First, our control group was significantly older than our PD group. While apathy is prevalent in numerous psychiatric conditions, it has also been described to increase with age within the healthy population (Brodaty, Altendorf, Withall, & Sachdev, 2010), and in particular has been associated with changes to frontal‐temporal structures that are observed with normal aging (Grool et al., 2014). Therefore, it is possible that the relatively high levels of apathy that we observed within our control group arose as a result of this increased age, which in turn may have dampened the true between‐group effect of total apathy. Although we utilized a reasonably rigorous recruitment protocol for this study, not all people contacted responded, and this may have biased our results, particularly if more apathetic people with less inclined to take part in the study. Finally, we administered the AMI directly to people with PD, rather than to an informant. There are pros and cons to using self‐ versus informant‐report scales when assessing apathy in PD (Dujardin et al., 2007), but there remains a possibility that apathy symptoms may have been underestimated in the clinical population in this study.

## 5 CONCLUSIONS

This work provides further evidence for a distinct pattern of apathy in people with PD in which deficits manifest in some—but not all—dimensions of motivated behavior. It emphasizes the importance of considering apathy as a multidimensional construct in clinical and research settings. Understanding how these dimensions relate to alterations in the neurobiological systems that underlie normal behavior remains a crucial research priority, as the field attempts to find effective treatments for this debilitating problem.

## CONFLICT OF INTEREST

The authors declare no conflict of interest.

### PEER REVIEW

The peer review history for this article is available at https://publons.com/publon/10.1002/brb3.2862

## Supporting information

Supporting Information

## Data Availability

Data used in this analysis is available upon reasonable request to the authors.
